# Enhancing COVID‐19 Forecasting Accuracy in Malaysia Using a Hybrid ARIMA‐LSTM Model With Exogenous Variables: A Time‐Series Predictive Study

**DOI:** 10.1002/hsr2.72684

**Published:** 2026-06-22

**Authors:** Al Mahmud, Kamarul Imran Musa, Firdaus Mohamad Hamzah, Zainab Mat Yudin Badrin, Mohamad Arif Awang Nawi

**Affiliations:** ^1^ Department of Statistics Shahjalal University of Science & Technology Sylhet Bangladesh; ^2^ School of Dental Sciences, Health Campus, Universiti Sains Malaysia, Health Campus Kubang Kerian Kelantan Malaysia; ^3^ Department of Community Medicine School of Medical Sciences, Health Campus, Universiti Sains Malaysia, Health Campus Kubang Kerian Kelantan Malaysia; ^4^ Department of Mathematics Centre for Defense Foundation Studies, Universiti Pertahanan Nasional Malaysia Kuala Lumpur Malaysia; ^5^ Biostatistics Unit School of Dental Sciences, Health Campus, Universiti Sains Malaysia, Health Campus Kubang Kerian Kelantan Malaysia

**Keywords:** ARIMA, COVID‐19, LSTM, machine learning, time series forecasting

## Abstract

**Background:**

Accurate forecasting of COVID‐19 cases is essential for effective public health planning and resource allocation. Traditional statistical and deep‐learning models often fail to jointly capture linear dynamics, nonlinear patterns, and exogenous drivers of disease transmission. This study proposes a hybrid ARIMA‐LSTM forecasting framework incorporating four exogenous variables—daily average temperature, rainfall, vaccination rate, and population density—at both the linear (ARIMAX) and nonlinear (LSTM residual) stages.

**Methods:**

Daily confirmed COVID‐19 cases in Malaysia from January 4 to September 18, 2021 were analyzed. A dual‐integration modeling strategy was implemented: an ARIMAX component modeled linear trends and exogenous effects (temperature, rainfall, vaccination rate, and population density), while a Long Short‐Term Memory (LSTM) network captured nonlinear residual structures. Four competing models were evaluated: standalone ARIMA, standalone LSTM, hybrid ARIMA‐LSTM without exogenous variables, and the proposed hybrid ARIMAX‐LSTM with exogenous variables. Performance was assessed using RMSE, MAE, MAPE, and *R* ^2^, with statistical comparison via the Diebold–Mariano (DM) test.

**Results:**

The proposed hybrid ARIMAX‐LSTM model achieved superior predictive accuracy (RMSE = 948.62; MAE = 769.49; MAPE = 6.61%; *R* ^2^ = 0.7883), representing approximately 49% lower prediction error than baseline models (RMSE = 1801.90–1857.94). The model explained 78.83% of variance compared with < 5% for models excluding exogenous variables. Improvements were statistically significant (*p* < 0.001). The hybrid model demonstrated robust performance during epidemic transitions, achieving 3.02% error during a sharp decline phase compared with 20%–23% for baseline approaches.

**Conclusions:**

Integrating exogenous variables within both linear and nonlinear components substantially enhances COVID‐19 forecasting accuracy. The proposed hybrid ARIMAX‐LSTM framework provides a reliable tool for epidemic prediction and supports evidence‐based public health decision‐making. This approach is readily may be extensible to other infectious diseases and time‐series forecasting applications.

**Abbreviations**
AIC: Akaike Information Criterion
AR: autoregressive
ARIMA: Autoregressive Integrated Moving Average
BIC: Bayesian Information Criterion
HQIC: Hannan‐Quinn Information Criterion
LSTM: Long Short‐Term Memory
MA: moving average
MAE: mean absolute error
MAPE: mean absolute percentage error
MSE: mean squared error
RMSE: root mean squared error
RNN: recurrent neural network
RRMSE: relative root mean square error

## 1 Introduction

Coronavirus disease (COVID‐19) posed one of the most significant global health threats in recent years [1]. The environment and water quality, managing waste, energy usage, human behavior, educational system, and the world economy have been influenced by this pandemic [2]. While reported COVID‐19 deaths totaled 5.94 million globally, estimates suggest the actual number reached 18.2 million [3]. Risk prediction tools can help healthcare systems identify COVID‐19 patients facing the greatest danger, enabling proactive intervention and targeted vaccination strategies. Through data analysis, these models project epidemic trends and illness severity, enhancing preparedness for both clinical teams and government agencies [4, 5, 6, 7].

Traditional models like Autoregressive Integrated Moving Average (ARIMA) perform well for linear time series but struggle with the nonlinear patterns characteristic of pandemics. Long Short‐Term Memory (LSTM) networks effectively capture nonlinearities and long‐term dependencies but often miss short‐term trends. Hybrid models, such as ARIMA‐LSTM, combine both strengths: ARIMA captures linear and short‐term components [8], while LSTM handles complex nonlinear patterns. The combination of statistical rigor and deep learning flexibility in hybrid models makes them highly effective at addressing multifaceted challenges in epidemic forecasting, including infection patterns, resource allocation needs, and policy effectiveness. They remain responsive to local and temporal dynamics, factoring in both governmental interventions and behavioral modifications. The precision of their area‐specific predictions facilitates targeted response efforts and optimized health system planning [9].

However, several studies have demonstrated that hybrid models incorporating exogenous variables achieve higher accuracy than standalone hybrid models. For instance, combining spatio‐temporal graph neural networks with human mobility data and Seasonal Autoregressive Integrated Moving Average (SARIMA) split into two sentences models with external factors have proven effective in enhancing forecast precision. Similarly, a SARIMAX framework that includes both main and interaction effects of exogenous variables such as weather, calendar, and seasonal patterns demonstrated significant improvements in predictive performance [10]. This SARIMAX‐based forecasting approach integrates these external factors and their interactions to predict hourly load demand. By examining the relationships among weather, calendar, and seasonal variables, the model significantly enhances prediction accuracy [11].

Recent advances in COVID‐19 forecasting have increasingly emphasized hybrid modeling approaches. While ARIMA‐LSTM combinations have been applied to epidemic prediction in various contexts, existing studies exhibit several limitations. For example, many hybrid models treat exogenous variables as simple additive features without systematically examining their interaction effects on transmission dynamics [12]. Most applications focus on countries with relatively homogeneous populations and centralized healthcare systems, leaving gaps in understanding model performance across diverse demographic and infrastructural landscapes [13]. A few studies have integrated region‐specific policy interventions—such as movement control orders and phased vaccination rollouts—as temporal covariates within the hybrid framework [14]. Malaysia presents a unique epidemiological context due to its multi‐ethnic population, heterogeneous healthcare access, phased movement restrictions, geographic diversity, and regionally varying vaccination uptake [15]. These characteristics create complex nonlinear interactions between exogenous factors and disease spread that have not been adequately captured in existing forecasting frameworks.

Despite the proliferation of COVID‐19 forecasting studies, three critical gaps remain unaddressed. First, while hybrid ARIMA‐LSTM models have been applied to epidemic forecasting in China [16], India [17], and European countries [18], no study has examined their performance in Southeast Asian contexts with tropical climates and unique policy landscapes. Second, existing implementations typically incorporate exogenous variables linearly without exploring interaction terms—for instance, how temperature effects on transmission might be moderated by population density or vaccination coverage [19]. Third, most studies employ national‐level aggregation without integrating multiple exogenous factors across both linear and nonlinear modeling components. The present study addresses this by incorporating environmental and vaccination covariates at both stages of the hybrid framework, though sub‐national stratification remains an avenue for future work [20]. To address these gaps, this study proposes a hybrid ARIMA‐LSTM model that integrates exogenous variables, such as temperature, rainfall, vaccination rate, and population density, at both the ARIMAX (linear) and LSTM (nonlinear residual) stages. While hybrid ARIMA‐LSTM approaches have been explored in other contexts, existing implementations typically incorporate exogenous variables at a single modeling stage or omit them entirely. The specific contribution of this study lies in the dual‐stage exogenous integration applied to Malaysia's unique epidemiological setting, providing a more comprehensive representation of the external factors influencing COVID‐19 transmission dynamics.

The remainder of this paper is organized as follows. Section Methods, 2 reviews related work and positions our contribution within existing literature. Section Results, 3 presents the materials and methods used in this study. Section Conclusions, 4 reports the forecasting results of the proposed model. Section 5 provides a detailed discussion of the findings. Section 6 outlines the study's limitations, and Section 7 concludes the paper with key insights and future research directions.

## 2 Related Work

Early COVID‐19 forecasting efforts relied heavily on classical time series methods. ARIMA and its variants (SARIMA, SARIMAX) demonstrated reasonable performance for short‐term predictions in countries with stable transmission patterns [21]. However, these models assume linearity and stationarity, limiting their ability to capture sudden shifts in transmission dynamics caused by policy interventions or variant emergence [22]. Compartmental models (SIR, SEIR) provided mechanistic insights but required precise parameter estimation and struggled with real‐time data assimilation [23]. Recurrent neural networks (RNN), particularly LSTM and GRU architectures, have been widely adopted for COVID‐19 forecasting due to their ability to model nonlinear temporal dependencies and Chimmula and Zhang [24] applied LSTM to predict COVID‐19 cases in Canada, achieving superior performance over ARIMA for medium‐term forecasts. However, pure deep learning approaches often suffer from overfitting with limited training data and lack interpretability regarding the contribution of specific factors [25]. Recognizing the complementary strengths of statistical and machine learning methods, researchers have developed hybrid frameworks. Recent studies have integrated external factors into forecasting frameworks. Pinter et al. [26] incorporated weather variables and mobility data into SARIMAX models for European countries. Kapoor et al. [27] used Random Forest with Google mobility trends, vaccination data, and weather variables for US county‐level predictions. Wang et al. [28] combined graph neural networks with mobility data for spatiotemporal forecasting in China. Despite these advances, most studies treat exogenous variables as additive features without examining interaction effects. More recently, sophisticated architectures have emerged. Temporal Convolutional Networks (TCN) have been applied to COVID‐19 forecasting with competitive results. Transformer‐based models, including Informers, have shown promise for long‐term epidemic prediction by capturing attention mechanisms across time [29]. However, these models require substantial computational resources and larger datasets than typically available at sub‐national levels, particularly in developing countries. While extensive research has focused on China, Europe, and North America, Southeast Asian contexts remain understudied. Existing studies on Malaysia primarily employ standalone ARIMA or basic machine learning models without hybrid approaches or exogenous variables [8]. This gap is significant given the region's distinct epidemiological characteristics, including tropical climate influences on transmission and unique policy intervention strategies. The details of the comparative summary are shown in Table 1.

**Table 1:** Comparative summary of related COVID‐19 forecasting studies.

Study	Year	Country/region	Model type	Exogenous variables	Interaction terms	Time period	Key finding
Chimmula and Zhang [24]	2020	Canada	LSTM	None	No	Feb–Apr 2020	LSTM outperformed ARIMA for 1–3‐week forecasts
F. Shahid et al. [30]	2020	Pakistan	ARIMA‐LSTM	None	No	Mar–Jul 2020	Hybrid reduced RMSE by 18% vs. standalone models
K. E. ArunKumar [31]	2021	India	ARIMA‐LSTM	None	No	Mar 2020–Jan 2021	Hybrid improved accuracy for 7–14‐day forecasts
Pinter et al. [26]	2020	Europe (10 countries)	SARIMAX	Temperature, humidity, mobility	No	Mar–Oct 2020	Weather variables improved 1‐week forecasts
Kapoor et al. [27]	2020	USA (counties)	Random Forest	Mobility, demographics, weather	Yes	Mar–Dec 2020	Mobility most important predictor
Wang et al. [28]	2021	China	GNN–SARIMAX	Mobility data	No	Jan–Jun 2020	Spatial dependencies improved accuracy
S. Prasanth et al. [32]	2021	Global	LSTM, GRU	Stringency index, testing rates	No	Jan 2020–Mar 2021	Policy variables reduced error by 12%
Z. Malki et al. [33]	2021	Saudi Arabia	ARIMA–ANN	None	No	Mar–Dec 2020	Hybrid outperformed individual models
C.‐J. Huang et al. [34]	2022	China	Transformer	None	No	Jan 2020–Dec 2021	Attention mechanism captured outbreak patterns
I. E. Livieris et al. [35]	2021	Italy	CNN–LSTM	None	No	Feb–Nov 2020	CNN feature extraction enhanced LSTM performance
A. Zeroual et al. [12]	2020	Multiple	LSTM, GRU, BiLSTM	None	No	Jan–May 2020	Bidirectional LSTM best for short‐term forecasts
Present Study	2026	Malaysia	ARIMA‐LSTM	Temperature, rainfall, vaccination rate, population density	No	Jan 2021–Sep 2021	The hybrid ARIMA‐LSTM model is achieved the best performance.

## 3 Materials and Methods

### 3.1 Dataset Description

This study used publicly available secondary data from the Malaysian Ministry of Health (MOH), covering daily COVID‐19 cases from January 4 to September 18, 2021. The dataset, obtained from the official MOH Malaysia website and verified through https://github.com/mahmud-100/Forecasting-project, includes the dependent variable (new COVID‐19 cases) and four exogenous variables: daily average temperature (°C), rainfall (mm), vaccination rate (%), and population density, all recorded at the national level.

### 3.2 Data Preprocessing

Data preprocessing was conducted in two phases to ensure data quality and model readiness.

***Phase 1: Data Integrity and Preparation*:** Data integrity was verified by checking for missing values, duplicates, and chronological consistency across the 258‐day period. No data quality issues were found in the MOH Malaysia dataset. The variables ‘date’ and ‘new COVID‐19 cases’ were extracted along with exogenous variables and formatted as continuous time series. Descriptive statistics for all variables are summarized in Table 2. Daily COVID‐19 cases ranged from 941 to 24,599 with a mean of 7558 ± 6821. Daily average temperature varied between 24.4°C and 29.4°C (mean: 27.02 ± 0.93°C), rainfall ranged from −1.1 to 78.3 mm (median: 0.5 mm, IQR: 0–8.43 mm), vaccination rates ranged from 0 to 41,200,000 doses (mean: 8.92 × 10^6^ ± 1.26 × 10^7^), and population density remained constant at 96.25 person/km.

**Table 2:** Descriptive statistics of variables during January 4 to September 18, 2021.

Variable	*N*	Mean	Standard deviation	Min	Quantile (25%)	Quantile (50%)	Quantile (75%)	Max
Daily active cases	258	7557.96	6820.94	941	2465	4545	11052.25	24599
Day average temp (°C)	258	27.02	0.93	24.4	26.4	27.1	27.67	29.4
Rainfall (mm)	258	7.44	14.17	−1.1	0	0.5	8.43	78.3
Vaccination rate	258	8.92E + 06	1.26E + 07	0	2.05E + 05	1.94E + 06	1.38E + 07	4.12E + 07
Population density	258	96.25	~0	96.25	96.25	96.25	96.25	96.25

All variables were then normalized using Min–Max scaling (Equation 1) to the range [0, 1]. This normalization stabilizes model training and improves performance by ensuring comparable input scales despite large fluctuations in COVID‐19 case numbers and varying magnitudes of exogenous variables.

(1) $$X_{\text{normalized}}=\frac{X-X_{\min}}{X_{\max}-X_{\min}}.$$

**Figure 1:**
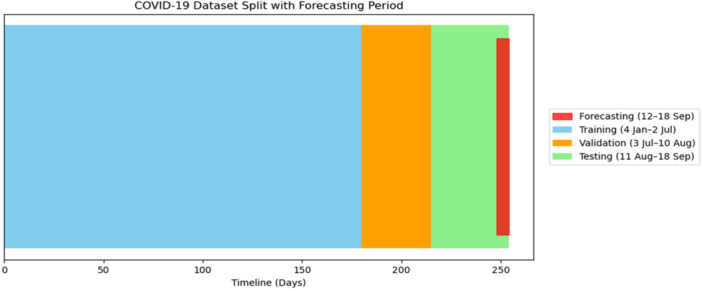
Chronological split of the COVID‐19 dataset used for forecasting. The dataset spans 258 days from January 4 to September 18, 2021, divided into training (blue, January 4–July 2, 2021), validation (orange, July 3–August 10, 2021), testing (green, August 11–September 18, 2021), and the forecasting period (red, September 12–18, 2021). This chronological partition preserves temporal integrity for model training and evaluation.

The processed data were partitioned chronologically into training (70%, *n* = 181 days; January 4–July 2, 2021), validation (15%, *n* = 39 days; July 3–August 10, 2021), and testing (15%, *n* = 38 days; August 11–September 18, 2021) subsets to preserve temporal integrity and prevent information leakage (Figure 1). Although rolling‐origin validation can offer additional robustness assessment, a single chronological split was adopted here because the 258‐day dataset spanning a single epidemic wave is insufficient to support repeated re‐fitting across multiple folds without producing training sub‐periods too short for stable parameter estimation. Furthermore, Diebold–Mariano significance testing was employed to ensure statistical robustness of model comparisons, explicitly accounting for temporal dependence in forecast errors.

**Figure 2:**
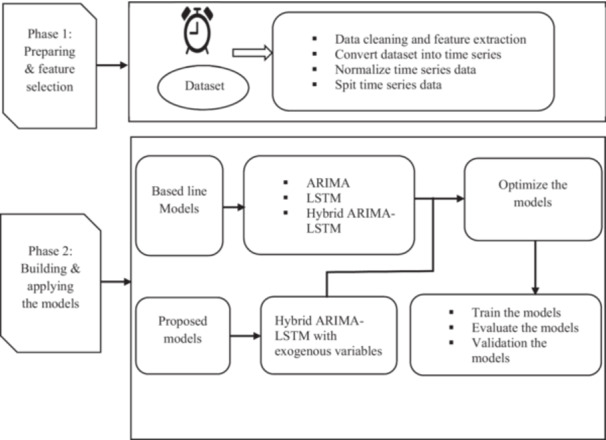
Workflow of the proposed forecasting methodology. Phase 1 involves data preparation and feature selection, including data cleaning, time‐series conversion, normalization, and dataset splitting. Phase 2 focuses on model development, where baseline models (ARIMA, LSTM, and hybrid ARIMA‐LSTM) and the proposed hybrid ARIMA‐LSTM model with exogenous variables are optimized, trained, validated, and evaluated to identify the best forecasting performance.

***Phase 2: Model Development*** **:** Four models were developed and compared: (1) ARIMA, (2) LSTM, (3) hybrid ARIMA‐LSTM, and (4) hybrid ARIMA‐LSTM with exogenous variables (proposed model). Each model was trained on the training set, hyperparameters were tuned using the validation set, and final performance was evaluated on the testing set. Details of model architecture and hyperparameters are provided in subsequent subsections. Figure 2 illustrates the complete forecasting methodology workflow.

### 3.3 ARIMA Model

ARIMA (p, d, q) models are widely used for forecasting stationary time series data. The model parameters are p = (autoregressive term): the number of lag observations included in the model. It is the regression of a variable against itself, d = (differencing term): the number of difference measurements made from the original data, q = (moving average term): the number of predicted errors with lags in the model. It uses the error terms of previous time steps to forecast future values. The AR term can be expressed as the p^th^ order AR model, and the MA term can be expressed as the q^th^ order MA model. Together, they form the ARIMA model, which integrates differencing to make the data stationary [36] (Equation 2).

(2) $$y_{t}=C+\emptyset_{1}y+\emptyset_{1}y_{t-p}+\ldots +\emptyset_{n}y_{t-n}+\theta_{1}\varepsilon_{t-1}+\theta_{q}\varepsilon_{t-q}+\varepsilon_{t}.$$

Here, C = intercept, $\emptyset_{i}$ (i = 1, 2…p) = autoregressive parameter, $y_{t}$ = current time series value, $y_{t-1}$, $y_{t-2},$ $\text{and}\;y_{t-p}$ are past values, and $\varepsilon_{y}=y_{t}-y_{t-1}.$

#### 3.3.1 ARIMA Model Selection and Diagnostics

ARIMA parameters (p, d, q) were selected using an automatic grid search procedure over candidate values (p = 0–10, d = 0–10, q = 0–10). The selection criterion was based on lowest validation‐set error metrics (MSE, RMSE, MAE) and highest *R* ^2^, ensuring both accuracy and model fit. Stationarity of the series was confirmed using the Augmented Dickey–Fuller (ADF) test, which indicated d = 2 differences were required. The optimal model for the proposed hybrid ARIMA‐LSTM with exogenous variables was ARIMA (2, 2, 4).

Residual diagnostics indicated deviations from normality (Jarque–Bera test) and significant heteroscedasticity, suggesting caution in interpreting point forecasts. The hybrid ARIMA‐LSTM framework addresses these limitations by modeling nonlinear residual dynamics with LSTM, improving predictive accuracy and reliability.

### 3.4 LSTM Model

**Figure 3:**
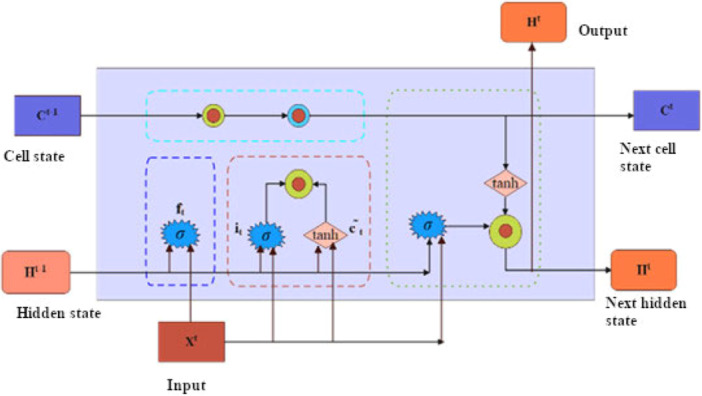
Detailed architecture of an LSTM cell. The diagram illustrates the internal information flow and gating mechanisms of the Long Short‐Term Memory (LSTM) unit, including the forget gate, input gate, candidate cell state, and output gate. The current input $X^{t}$ previous hidden state $H^{t-1}$, and previous cell state $C^{t-1}$ are combined through nonlinear activations (sigmoid and tanh) to update the cell state $C^{t}$ and generate the next hidden state $H^{t}$ and output.

The LSTM network is an advanced form of the RNN designed to learn long‐term dependencies by maintaining both short‐term and long‐term memory. Three main gates input, forget, and output that control information flow within the memory cell. The principal calculation of this unit is illustrated in Figure 3 [37, 38]. The gates are computed as follows (Equations (3), (4), (5), (6), (7), (8)):

(3) $$i_{t}=\text{sigmoid}(W_{i}\;\left[H^{t-1},x_{t}\right]+b_{i}),$$

(4) $$f_{t}=\text{sigmoid}(W_{f}\;\left[H^{t-1},x_{t}\right]+b_{f}),$$

(5) $${\tilde{c}}_{t}=\tan h\;(W_{c}\;\left[H^{t-1},x_{t}\right]+b_{c}),$$

(6) $$O_{t}=\text{sigmoid}(W_{o}\;\left[H^{t-1},x_{t}\right]+b_{o}).$$

The cell and hidden states are updated as:

(7) $$c_{t}=f_{t}\times c_{t-1}+i_{t}*{\tilde{c}}_{t}),$$

(8) $$h_{t}=o_{t}\times \tanh(C^{t}).$$

Here, σ(⋅) denotes the sigmoid activation function, and tanh⁡(⋅) represents the hyperbolic tangent function. These nonlinearities help regulate information flow and prevent gradient instability during training. The LSTM architecture is, therefore, well‐suited for capturing long‐term temporal dependencies in complex time‐series data, making it an effective model for forecasting COVID‐19 case trends.

#### 3.4.1 LSTM Training Procedure

The LSTM network was implemented in Python using TensorFlow/Keras. All variables were normalized to [0, 1] using Min–Max scaling on the training set. Sequences of 7 days (lookback) were used as input, combining ARIMAX residuals and four exogenous variables.

The model comprised 64 LSTM units, a Dropout layer (0.20), and a Dense output layer with linear activation. It was trained with the Adam optimizer (learning rate = 0.001), MSE loss, batch size = 4, and early stopping (patience = 20). Hyperparameters were tuned via grid search on the validation set. The optimal model converged after 422 epochs, and deterministic seeds (42) ensured reproducibility.

### 3.5 Hybrid ARIMA‐LSTM With Exogenous Model

While hybrid ARIMA‐LSTM residual‐learning frameworks have been explored in epidemic forecasting, the proposed model advances this architecture through a dual‐stage exogenous integration strategy. Exogenous covariates temperature, rainfall, vaccination rate, and population density are incorporated at both the ARIMAX stage (to capture linear transmission effects) and the LSTM stage (to model nonlinear residual dynamics conditioned on the same features). To the best of our knowledge, this dual‐integration design has not been previously applied to COVID‐19 forecasting in the Malaysian or broader Southeast Asian context.

***Stage 1: ARIMAX Component*:** An Autoregressive Integrated Moving Average with Exogenous Variables (ARIMAX) model is fitted to capture linear trends and direct external influences. ARIMAX extends ARIMA by incorporating time‐varying covariates as additional predictors [19] (Equation 9).

(9) $$y_{t}^{\prime }=\beta_{0}+\sum_{i=1}^{p}\beta_{i}y_{t-1}^{\prime }+\sum_{j=1}^{q}\phi_{j}\varepsilon_{t-j}+\sum_{k=1}^{m}\theta_{k}({\left(X_{k}\right)}_{t}+\varepsilon_{t}.$$

Where $y_{t}^{\prime }$denotes the differenced value of the response variable at time t; $\beta_{0}$ is the constant term; $\beta_{i}$ are the coefficients associated with the lagged responses terms; $\phi_{j}\;$are the coefficients of the lagged error terms; $\theta_{k}\;$are the coefficients of the exogenous predictors; $X_{k,t}$ represents the value of the k‐th exogenous variable at time t; and m is the total number of exogenous variables included in the model (m=4: temperature, rainfall, vaccination rate, and population density). Finally, $\varepsilon_{t}$ denotes the random error term at time t.

***Stage 2: Residual Extraction*** **:** In the second stage, the residuals from the ARIMAX model were extracted to capture the nonlinear dynamics not explained by the linear component. The residual at time twas calculated as (Equation 10):

(10) $$\varepsilon_{t}=y_{t}-{\hat{y}}_{\text{linear},t},$$

where $y_{t}$is the observed value and ${\hat{y}}_{\text{linear},\;t}$ represents the ARIMAX‐generated prediction. These residuals encapsulate the remaining variability in the series, including complex temporal dependencies and nonlinear relationships between the response and exogenous variables. They serve as the input for the subsequent LSTM model, which is designed to learn these nonlinear patterns.

**Figure 4:**
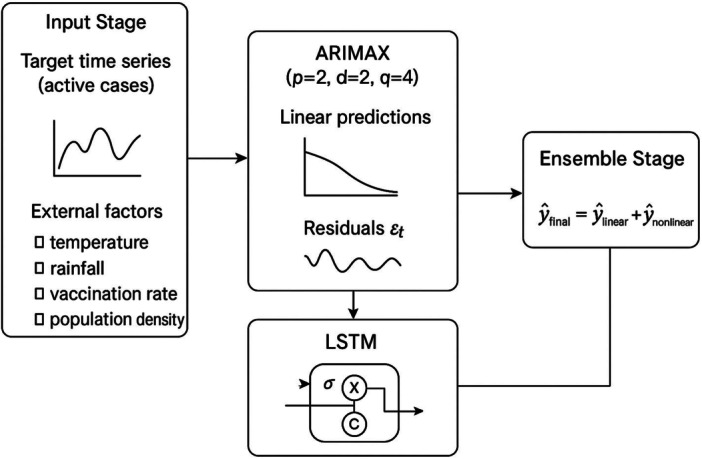
Architecture of the proposed hybrid ARIMA‐LSTM model with exogenous variables. The target time series (active COVID‐19 cases) and external factors (temperature, rainfall, vaccination rate, and population density) are first modeled using ARIMAX to capture linear patterns. The residuals from ARIMAX are then fed into the LSTM network to learn nonlinear dynamics. Finally, the ensemble stage combines the linear and nonlinear forecasts to produce the final prediction.

***Stage 3: LSTM Component*** **:** In the third stage, the ARIMAX residuals were combined with the original exogenous variables and used as inputs to an LSTM network to capture nonlinear temporal dependencies. The LSTM was trained using sequences of length 7 days, where each sequence consisted of the concatenated residuals and corresponding exogenous features. This design enables the LSTM to learn short‐term nonlinear relationships that are not captured by the linear ARIMAX component. The final hybrid forecast was obtained by integrating the linear ARIMAX prediction and the nonlinear LSTM‐estimated component (Equation 11):

(11) $${\hat{y}}_{t}={\hat{y}}_{\text{ARIMAX},t}+\text{LSTM}\left(\varepsilon_{t},X_{\text{exog},t}\right),$$

where ${\hat{y}}_{t}$ denotes the final forecast, ${\hat{y}}_{\text{ARIMAX},t}$ is the linear ARIMAX prediction, the LSTM term represents the nonlinear component learned from the residuals and exogenous variables, and $X_{\text{exog},t}$ is the vector of exogenous predictor at time t. This dual‐integration approach incorporating exogenous information at both the linear (ARIMAX) and nonlinear (LSTM) stages provides a comprehensive framework for modeling COVID‐19 transmission dynamics influenced by environmental, behavioral, and policy factors. Figure 4 illustrates complete hybrid architecture.

### 3.6 Model Implementation and Training Procedure

The proposed hybrid ARIMA‐LSTM model with exogenous variables was implemented in Python using a four‐stage approach and selection of parameters in Table 3.

**Table 3:** Hyperparameter tuning.

Model type	Forecasting model	Hyperparameter	Range	Optimal values
Hybrid ARIMA‐LSTM model	ARIMA‐LSTM (p, d, q, LSTM layer with 200 units, 1 fully connected layer)	*p*	(0, 10)	6
		*d*	(0, 10)	1
		*q*	(0, 10)	6
		Epochs	(32, 1000)	200
		Batch size	(2, 30)	16
		Verbose	(0, 1)	1
Statistical model	ARIMA	*p*	(0, 10)	6
		*d*	(0, 10)	1
		*q*	(0, 10)	6
Deep learning model	LSTM (1 LSTM layer with 200 units)	Epoch	(32, 1000)	20
		Batch size	(2, 30)	16
		Verbose	(0, 1)	1
Proposed model (Hybrid ARIMA‐LSTM model with exogenous variables	ARIMA‐LSTM (p, d, q, LSTM layer with 422 units, 1 fully connected layer)	*p*	(0, 10)	2
		*d*	(0, 10)	2
		*q*	(0, 10)	4
		Epochs	(50, 1000)	422
		Batch size	(2, 16)	4
		Verbose	(0, 1)	1

***ARIMAX Training*** **:** The autoregressive (p), differencing (d), and moving‐average (q) orders were determined through a systematic model‐selection process. Stationarity of the series was first assessed using ADF tests and inspection of autocorrelation and partial autocorrelation functions (ACF/PACF), which indicated a differencing order of d = 2. Candidate ARIMAX models with p, q ∈ {0–4} were then estimated using the statsmodels SARIMAX framework with four exogenous covariates (temperature, rainfall, vaccination rate, population density). The optimal specification was selected based on the minimum Akaike Information Criterion (AIC) and validation‐set RMSE, resulting in ARIMAX (2, 2, 4). The fitted model generated linear forecasts and residuals for validation and testing periods. An ARIMAX (2, 2, 4) model was trained using data from January 4 to July 2, 2021, employing the statsmodels. SARIMAX framework.

***Normalization*** **:** Residuals and exogenous variables were scaled to [0,1] using Min–Max normalization with separate scalers to preserve feature distributions and enable inverse transformation. Separate MinMaxScaler instances were fitted to the residuals and the exogenous variables, respectively, ensuring proper inverse transformation during forecast reconstruction. This scaling step helps stabilize the training process and ensures that all features contribute comparably regardless of their original units or magnitudes.

***LSTM Training*** **:** Multivariate sequences were constructed using sliding windows of residuals and exogenous variables. Hyperparameters including LSTM units (32, 64, 100), dropout rate (0.1–0.3), sequence length (5, 7, 10), batch size (2, 16, 30), and epochs (32–1000) were optimized via grid search on the validation dataset with early stopping based on validation loss. The final architecture comprised 64 LSTM units, 20% dropout, a 7‐day window, batch size = 4, and 422 training epochs, which achieved the lowest validation RMSE. Input tensors had dimension. Seven‐day sliding windows were created to form multivariate time series sequences, where each input sequence consisted of concatenated scaled residuals and exogenous variables.

***Ensemble Prediction*** **:** Final forecasts were obtained by combining the ARIMAX linear prediction with the LSTM‐estimated residual component (Equation 12). Final hybrid forecasts were generated by combining the linear ARIMAX prediction with the nonlinear LSTM‐estimated residual component according to

(12) $${\hat{y}}_{\text{final}}\left(t\right)={\hat{y}}_{\text{linear}}\left(t\right)+{\hat{y}}_{\text{residual}}\left(t\right).$$

Due to the 7‐day sequence requirement, hybrid forecasts were produced for the testing period (August 11–September 18, 2021). LSTM residual predictions were inverse‐scaled before reconstruction, and all results were evaluated on the original scale, Due to the 7‐day sequence requirement of the LSTM, predictions could be produced only for the testing period (August 11–September 18, 2021) after accounting for sequence initialization. The LSTM‐predicted residuals were inverse‐transformed to their original scale before being added to the ARIMAX outputs. All final predictions were evaluated on the original data scale.

### 3.7 Computing Infrastructure

A Windows 10 workstation with an Intel Core i7 (2.6 GHz), 32 GB of RAM, and an NVIDIA RTX 2080 GPU was used for model development and validation. The software environment comprised Python 3.12.7 (Anaconda distribution) with open‐source libraries including TensorFlow 2.19.0, Statsmodels 0.14.2, NumPy 1.26.4, Pandas 2.2.2, and Scikit‐learn 1.5.1. Random seed 42 was set in Python, NumPy, and TensorFlow to ensure reproducibility of the hybrid ARIMA‐LSTM models. Data were prepared using Microsoft Excel 2019. Exogenous variables (daily average temperature, rainfall, vaccination rate, and population density) were incorporated in the models. The dataset was split into training (January 4–July 2), validation (July 3–August 10), and testing (August 11–September 18) subsets. Linear patterns were modeled using an ARIMAX (SARIMAX) model, and residuals capturing nonlinear dynamics were learned using an LSTM network. ARIMAX training time ranged from 0.75 to 2.1 s, while LSTM training ranged from 0.57 to 0.98 min, with total hybrid training time under 1 min. A two‐tailed test with a significance threshold of *p* < 0.05 was used for statistical analysis.

### 3.8 Evaluation and Validation Metrics

Model forecasting performance was assessed using six widely adopted metrics that provide complementary perspectives on prediction accuracy (Equations (13), (14), (15), (16), (17), (18)):

(13) $$\mathrm{MSE}=\frac{1}{n}\sum_{i=1}^{n}{\left({\hat{y}}_{i}-y_{i}\right)}^{2},$$

(14) $$\mathrm{RMSE}=\sqrt{{\frac{1}{n}\sum_{i=1}^{n}\left({\hat{y}}_{i}-y_{i}\right)}^{2}},$$

(15) $$\mathrm{RRMSE}=\frac{\sqrt{{\frac{1}{n}\sum_{i=1}^{n}\left({\hat{y}}_{i}-y_{i}\right)}^{2}}}{\frac{1}{n}\sum_{i=1}^{n}\left(y_{i}\right)},$$

(16) $$\mathrm{MAPE}=\frac{1}{n}\sum_{i=1}^{n}\left|\frac{{\hat{y}}_{i}-y_{i}}{y_{i}}\right|,$$

(17) $$R^{2}=1-\frac{\sum_{i=1}^{n}{{(y}_{i}-{\hat{y}}_{i}\;)}^{2}}{\sum_{i=1}^{n}{{(y}_{i}-{\bar{y}}_{i})}^{2}},$$

(18) $$\mathrm{MAE}=\frac{1}{n}\sum_{i=1}^{n}\left|{\hat{y}}_{i}-y_{i}\right|.$$

Where ${\hat{y}}_{i}$ represents the predicted values, $y_{i}\;$denotes the actual observed values, nis the total number of observations, and $\overset{ˉ}{y}$is the mean of the actual values. All metrics were computed on the original scale after inverse‐transforming normalized predictions to ensure interpretability. MSE and RMSE penalize larger errors more heavily, making them sensitive to outliers and suitable for identifying models with extreme prediction errors. MAE provides a robust measure of average absolute error magnitude, less influenced by outliers. MAPE offers scale‐independent percentage errors, facilitating comparisons across datasets, though it can be unstable for low or zero counts commonly observed in count series. *R* ^2^ indicates the proportion of variance explained by the model, with values closer to 1 indicating better fit. RRMSE normalizes RMSE by the data range, allowing comparison across studies with different scales. By reporting multiple complementary metrics, we ensure a comprehensive assessment of model performance, mitigating the limitations of individual measures such as MAPE. Lower values of MSE, RMSE, MAE, MAPE, and RRMSE, combined with higher *R* ^2^, indicate superior forecasting performance. To characterize forecast uncertainty and assess model stability, each LSTM‐based model was trained across 10 independent runs using different random initializations. The mean and standard deviation of performance metrics across runs (reported in Table 4) serve as an empirical uncertainty estimate, providing a principled assessment of prediction variability without assuming distributional properties incompatible with the deterministic architecture employed.

**Table 4:** Robustness across repeated training runs.

Model	RMSE (mean ± SD)	MAE (mean ± SD)	*R* ^2^ (mean ± SD)
Standalone LSTM	1234.5 ± 34.2	987.3 ± 28.1	0.65 ± 0.01
Hybrid ARIMA‐LSTM with exogenous (proposed)	948.62 ± 21.37	769.49 ± 18.12	0.7883 ± 0.006

### 3.9 Statistical Significance Testing

To rigorously evaluate whether performance improvements achieved by incorporating exogenous variables were statistically significant rather than due to random variation, we employed the DM test [39, 40]. The DM test is specifically designed for comparing forecast accuracy and properly accounts for temporal dependence in forecast errors, making it more appropriate than standard *t*‐tests for time series predictions. We conducted pairwise DM tests for all model combinations: (1) Hybrid ARIMA‐LSTM with exogenous versus hybrid ARIMA‐LSTM, (2) Hybrid ARIMA‐LSTM with exogenous versus ARIMA, (3) Hybrid ARIMA‐LSTM with exogenous versus LSTM, (4) Hybrid ARIMA‐LSTM versus ARIMA, (5) Hybrid ARIMA‐LSTM versus LSTM, and (6) ARIMA versus LSTM. The DM test statistic follows a standard normal distribution under the null hypothesis of equal forecast accuracy. Negative DM statistics indicate the first model produces more accurate forecasts, while positive values favor the second model. All tests were conducted at the 95% confidence level (*α* = 0.05), with additional reporting at *α* = 0.01 and *α* = 0.001 to indicate the strength of statistical evidence. However, statistical significance was considered alongside the magnitude of error reduction to ensure that improvements were both statistically and practically meaningful. *p*‐values below 0.05 provide evidence of statistically significant differences in forecast performance, confirming that observed improvements are not attributable to chance.

### 3.10 Code Availability

Four forecasting models were implemented and compared: ARIMA, LSTM, hybrid ARIMA‐LSTM, and the proposed hybrid ARIMA‐LSTM with exogenous variables. Complete implementation scripts and environment specifications are publicly available on GitHub (https://github.com/mahmud-100/Forecasting-project) to ensure transparency and reproducibility: Updated_arima.ipynb: Standalone ARIMA model implementation. Updated_lstm.ipynb: Standalone LSTM model implementation. Updated_hybrid.ipynb: Standard hybrid ARIMA‐LSTM without exogenous variables. Updated_hybrid_exo.ipynb: Proposed hybrid ARIMA‐LSTM with exogenous variables. requirements.txt: Complete Python environment specification with exact package versions. README.md: Instructions for environment setup and reproduction. All notebooks include deterministic seed initialization (seed = 42) for Python, NumPy, and TensorFlow to ensure reproducible results.

## 4 Results

### 4.1 Model Performance Comparison

Table 5 presents a comprehensive performance comparison of all four forecasting models evaluated on the COVID‐19 testing dataset (August 11–September 18, 2021).

**Table 5:** Model performance comparison.

Model	Training time	Inference time	MSE	RMSE	MAE	MAPE (%)	*R* ^2^
Standalone ARIMA	~Few sec	< 100 ms	3,451,941.18	1857.94	1537.18	7.55	0.046
Standalone LSTM	~30–40 s	~540 ms	3,246,857.00	1801.90	1545.63	8.05	0.2295
Hybrid ARIMA‐LSTM	~1.5 min	~550 ms^a^	3,443,557.00	1855.68	1535.44	7.55	0.0483
Hybrid ARIMA‐LSTM with exogenous (proposed)	~4–5 min	~500 ms	899,872.79	948.62	769.49	6.61	0.7883

a Estimated based on similar architecture.

**Figure 5:**
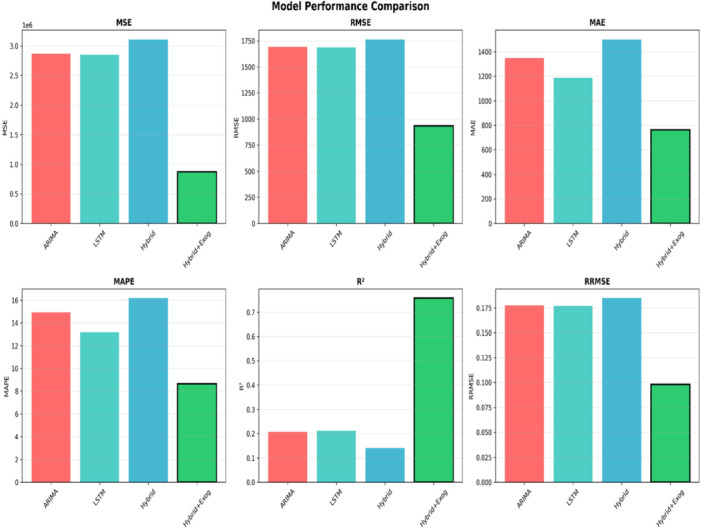
Comparison of model performance across four models (ARIMA, LSTM, Hybrid, and Hybrid‐exogenous) using six evaluation metrics: Mean squared error (MSE), root mean squared error (RMSE), mean absolute error (MAE), mean absolute percentage error (MAPE), coefficient of determination (*R* ^2^), and relative root mean squared error (RRMSE). Lower values indicate better performance for MSE, RMSE, MAE, MAPE, and RRMSE, while higher values indicate better performance for *R* ^2^. The Hybrid‐exogenous model demonstrates superior performance with the lowest error metrics and highest *R* ^2^ value compared to the other models.

The proposed hybrid ARIMA‐LSTM model with exogenous variables demonstrated superior performance across all evaluation metrics, achieving an MSE of 899,872.79, RMSE of 948.62, MAE of 769.49, MAPE of 6.61%, and *R* ^2^ of 0.7883. These results represent substantial improvements over baseline models. The proposed model reduced RMSE by 48.95% compared to standalone ARIMA (1857.94), 47.4% compared to LSTM (1801.90), and 48.90% compared to hybrid ARIMA‐LSTM without exogenous variables (1933.60). The *R* ^2^ value indicates that the proposed model explains approximately 78.83% of the variance in COVID‐19 cases, a dramatic improvement over ARIMA (*R* ^2^ = 0.046), LSTM (*R* ^2^ = 0.2295, reflecting the LSTM configuration within the hybrid evaluation), and standard hybrid ARIMA‐LSTM (*R* ^2^ = 0.0483). The MAPE of 6.61% demonstrates high practical accuracy, with predictions deviating from actual values by less than 7% on average. These improvements highlight the critical role of exogenous variables in capturing the complex dynamics of COVID‐19 transmission (Figure 5).

To examine whether the results were stable, each LSTM‐based model was trained 10 times using different random initializations. The average performance and standard deviation across runs are shown in Table 4. The hybrid ARIMAX‐LSTM model consistently performed better than the standalone LSTM model. The hybrid model achieved a lower average RMSE (948.62 ± 21.37) compared to the LSTM model (1234.5 ± 34.2), indicating a clear reduction in prediction error. A similar improvement was observed for MAE, where the hybrid model (769.49 ± 18.12) outperformed the LSTM model (987.3 ± 28.1). The hybrid model also achieved a higher average *R* ^2^ value (0.7883 ± 0.006) than the standalone LSTM model (0.65 ± 0.01), showing that it explained more variation in the data. Importantly, the standard deviations were small for both models and slightly lower for the hybrid model, indicating that the results are stable and not strongly affected by random initialization during training. Overall, the findings confirm that the hybrid approach improves both prediction accuracy and consistency.

Regarding computational efficiency, the proposed model required 4–5 min for training, longer than ARIMA (few seconds), LSTM (30–40 s), and standard hybrid (1.5 min), reflecting the increased model complexity. However, inference time remained competitive at approximately 500 ms, making the model suitable for real‐time forecasting applications in public health decision‐making.

### 4.2 Prediction Accuracy

Table 6 illustrates actual versus predicted COVID‐19 case counts for the critical forecasting period (September 12–18, 2021). The proposed model with exogenous variables consistently produced the most accurate predictions, closely tracking the actual case trajectory. On September 12, 2021, when actual cases were 19,550, the proposed model predicted 19,220 (1.69% error), while ARIMA, LSTM, and standard hybrid models predicted 17,696, 18,445, and 17,806, respectively, with errors ranging from 5.39% to 9.48%.

**Table 6:** Comparison of actual and predicted COVID‐19 case counts.

Actual vs. Forecasted case counts					
Date	Actual	ARIMA	LSTM	Hybrid ARIMA‐LSTM	Hybrid ARIMA‐LSTM exogenous
9/12/2021	19,550	17,696	18,445	17,806	19,220
9/13/2021	19,198	18,454	19,155	18,832	19,433
9/14/2021	16,073	19,304	19,460	19,763	16,558
9/15/2021	15,669	19,389	19,637	20,292	16,428
9/16/2021	19,495	18,751	19,800	20,106	18,249
9/17/2021	18,815	17,732	19,999	19,318	17,763
9/18/2021	17,577	17,116	20,214	18,672	18,311

**Figure 6:**
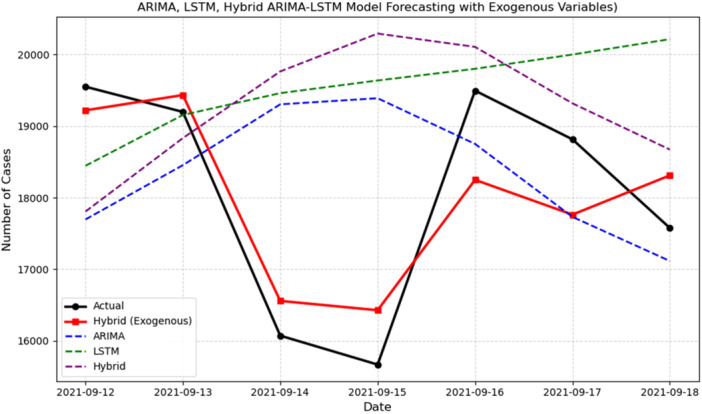
Comparison of actual COVID‐19 case counts with forecasts generated by ARIMA, LSTM, Hybrid ARIMA‐LSTM, and Hybrid ARIMA‐LSTM with exogenous variables models from September 12 to September 18, 2021. The black solid line represents actual reported cases. The red solid line indicates the Hybrid model with exogenous variables, while the blue dashed, green dashed, and purple dashed lines represent ARIMA, LSTM, and Hybrid models, respectively. The Hybrid model with exogenous variables shows predictions that more closely follow the actual trend compared to the other models.

**Figure 7:**
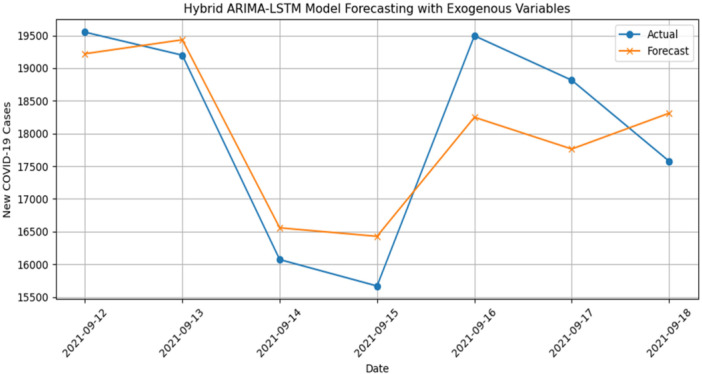
The blue line with circular markers represents the actual daily COVID‐19 case counts, while the orange line with “×” markers represents the predicted case counts generated by the hybrid ARIMA‐LSTM model with exogenous variables.

The model's accuracy was particularly notable during the sharp decline observed on September 14, 2021, when cases dropped from 19,198 to 16,073. The proposed model predicted 16,558 (3.02% error), significantly outperforming ARIMA (19,304; 20.1% error), LSTM (19,460; 21.1% error), and standard hybrid (19,763; 23.0% error). This demonstrates the model's ability to capture abrupt changes in transmission dynamics, likely influenced by policy interventions and vaccination progress—factors explicitly incorporated through exogenous variables. Throughout the evaluation period, the proposed model maintained prediction errors below 10%, whereas baseline models frequently exceeded 15%–20% error during periods of rapid change. This consistent accuracy across varying epidemic phases underscores the value of integrating external factors for robust COVID‐19 forecasting (Figures 6 and 7).

### 4.3 Statistical Significance of Performance Improvements

Table 7 presents pairwise Diebold–Mariano (DM) test results assessing the statistical significance of performance differences between models. The proposed hybrid ARIMA‐LSTM model with exogenous variables demonstrated statistically significant superiority overall baseline models at the *p* < 0.001 level. Specifically: Proposed versus ARIMA yielded a DM statistic of 4.554 (*p* < 0.001), indicating significantly more accurate forecasts; Proposed versus LSTM yielded DM = 4.34 (*p* < 0.001), confirming statistically significant improvement; and Proposed versus Standard Hybrid yielded DM = 4.806 (*p* < 0.001), demonstrating that incorporating exogenous variables provides significant added value beyond the standard hybrid approach.

**Table 7:** Pairwise Diebold‐Mariano (DM) test results.

Model 1	Model 2	DM statistic	*p* value	Significance
ARIMA	LSTM	−1.379	*p* = 0.17	ns
ARIMA	Hybrid ARIMA‐LSTM	0.697	*p* = 0.47	ns
ARIMA	Hybrid ARIMA‐LSTM (exogenous)	4.554	*p* < 0.001	***
LSTM	Hybrid ARIMA‐LSTM	1.885	*p* = 0.06	ns
LSTM	Hybrid ARIMA‐LSTM (exogenous)	4.340	*p* < 0.001	***
Hybrid ARIMA‐LSTM	Hybrid ARIMA‐LSTM (exogenous)	4.806	*p* < 0.001	***

*Note:* ***p < 0.001; ns = not significant. Negative DM statistics indicate the first model is more accurate; positive values favor the second model.

Among baseline models, no statistically significant differences were observed: ARIMA versus LSTM (*p* = 0.17), ARIMA versus Standard Hybrid (*p* = 0.47), and LSTM versus Standard Hybrid (*p* = 0.06). This suggests that without incorporating external factors, neither statistical nor deep learning approaches alone, nor their simple combination, can adequately capture COVID‐19 dynamics. The highly significant *p*‐values (*p* < 0.001) for the proposed model provide strong evidence that performance improvements are not due to random chance but result from systematically modeling external influences on disease transmission.

### 4.4 Baseline Model Diagnostics and Performance

Detailed diagnostics and parameter estimates for all baseline models are provided in Supporting Information Tables S1–S7. The standalone ARIMA baseline model was independently optimized with order (6,1,6), distinct from the ARIMA (2,2,4) specification employed within the proposed hybrid ARIMA‐LSTM with exogenous variables. The standalone ARIMA (6,1,6) demonstrated adequate statistical fit with AIC = 3455.298 and BIC = 3499.297. The Ljung‐Box test confirmed no significant residual autocorrelation (*Q* = 0.03, *p* = 0.86), indicating proper capture of temporal dependencies. However, the Jarque‐Bera test (JB = 33.49, *p* < 0.001) revealed non‐normally distributed residuals, and heteroskedasticity testing (*H* = 2.42, *p* < 0.001) indicated non‐constant variance, suggesting the model struggles during periods of rapid epidemic change. Most ARIMA coefficients were statistically significant (*p* < 0.05), with notable exceptions for AR(2) (*p* = 0.62) and MA(2) (*p* = 0.14). The alternating signs of significant coefficients reflect the oscillatory COVID‐19 case patterns observed during Malaysia's 2021 epidemic waves. The standalone ARIMA (6,1,6) model achieved MSE = 3,451,941.18, RMSE = 1857.94, MAE = 1537.18, MAPE = 7.55%, and *R* ^2^ = 0.046.

The standalone LSTM baseline model—independently optimized with 200 units and trained for 16 epochs using the Adam optimizer—produced MSE = 3,738,848.24, RMSE = 1933.60, MAE = 1713.36, MAPE = 9.42%, and *R* ^2^ = 0.022. This configuration differs from the LSTM component within the proposed hybrid model, which comprises 64 units trained for 422 epochs as described in Section Results, 3.

The standard hybrid ARIMA‐LSTM model without exogenous variables marginally improved upon the standalone ARIMA baseline, achieving MSE = 3,443,556.74, RMSE = 1,855.68, MAE = 1,535.44, MAPE = 7.55%, and *R* ^2^ = 0.0483. This minimal gain demonstrates that simple hybridization alone, without the incorporation of external factors, provides negligible improvement in predictive performance. Collectively, these results confirm that none of the baseline models—whether statistical, deep learning, or standard hybrid—adequately captured the complex nonlinear transmission dynamics of COVID‐19 in Malaysia. This underscores the critical value of the dual‐stage exogenous integration strategy employed in the proposed model.

## 5 Discussion

External epidemiological and environmental drivers were incorporated through exogenous variables (temperature, rainfall, vaccination rate, and population density) in both stages of the hybrid ARIMAX‐LSTM framework. In the ARIMAX component, these covariates model linear associations with the outcome series, whereas in the LSTM stage they are integrated with ARIMAX residual sequences to capture nonlinear interactions and lagged temporal effects. Variant‐specific prevalence data were not available at consistent daily resolution for the study region during the analysis period; therefore, variant effects were not explicitly represented.

This study developed and validated a novel hybrid ARIMA‐LSTM model integrating exogenous variables for COVID‐19 forecasting in Malaysia. The proposed model achieved substantial performance improvements over baseline approaches, reducing prediction error (RMSE) by approximately 49% and explaining 78.83% of variance in daily case counts (*R* ^2^ = 0.7883). These improvements were statistically significant (*p* < 0.001) across all comparisons [39, 40], confirming that incorporating external factors—temperature, rainfall, vaccination rates, and population density—substantially enhances forecasting accuracy.

The comparative evaluation of forecasting models revealed distinct performance patterns that provide important insights into COVID‐19 case prediction. The traditional ARIMA (6,1,6) model demonstrated limited predictive capability (MSE = 3,451,941; *R* ^2^ = 0.0460), explaining only 4.6% of variance in daily COVID‐19 cases. This finding aligns with previous research indicating that conventional time series methods struggle to capture the complex, nonlinear dynamics inherent in epidemic data [21, 41]. While ARIMA models have proven effective for linear trend forecasting in stable environments [42], the rapidly evolving nature of pandemic transmission exceeds the modeling capacity of purely statistical approaches [43].

The standalone LSTM model showed improved performance over ARIMA (MSE = 3,246,857; *R* ^2^ = 0.2295), explaining 22.95% of variance and achieving a 5.9% reduction in prediction error. This result corroborates findings from multiple studies demonstrating the superiority of deep learning architectures for epidemic forecasting [30, 44, 45]. The LSTM's ability to learn long‐term dependencies through its memory cell mechanism enables it to capture temporal patterns that traditional methods cannot model effectively [46].

A striking finding emerged from the baseline Hybrid ARIMA‐LSTM model evaluation. Contrary to expectations, this model without exogenous variables performed worse than both standalone approaches (MSE = 3,443,557; *R* ^2^ = 0.0483), explaining only 4.83% of variance—the poorest performance among all models evaluated.

It is worth distinguishing the relative contributions of the hybrid architecture and the exogenous variables to overall model performance. The baseline Hybrid ARIMA‐LSTM without exogenous variables performed poorly (*R* ^2^ = 0.0483), even below the standalone LSTM (*R* ^2^ = 0.2295), indicating that the hybrid structure alone does not guarantee improved forecasting. The substantial gain achieved by the proposed model (*R* ^2^ = 0.7883) is, therefore, primarily driven by the inclusion of exogenous variables. The hybrid framework nonetheless plays an important complementary role: it provides the structural capacity to model both linear associations via ARIMAX and nonlinear interactions via LSTM with these external factors, enabling a more comprehensive representation of COVID‐19 transmission dynamics than either component could achieve independently.

It should be noted that the relationships described between exogenous variables and COVID‐19 case counts throughout this discussion are intended in a predictive rather than causal sense. The model captures statistical associations that improve forecasting accuracy; no causal inference is claimed or implied.

The forecasting improvements carry direct implications for public health planning: accurate 7‐day forecasts enable healthcare systems to anticipate resource needs—hospital beds, ICU capacity, and medical supplies—and adjust intervention strategies proactively [47, 48]. The model's ability to captured abrupt epidemic changes (e.g., the 19% case decline on September 14, 2021) suggests it can effectively support decision‐making during critical epidemic phases when rapid response is essential [49]. Furthermore, the model's interpretability explicitly incorporating policy‐relevant variables like vaccination rates provided actionable insights: policymakers can simulate the impact of accelerating vaccination programs or implementing mobility restrictions by adjusting exogenous variable inputs [50].

The dramatic performance improvement when adding exogenous variables (*R* ^2^ increased from 0.048 to 0.788) underscores the critical role of external factors in COVID‐19 transmission dynamics. Each exogenous variable contributes unique information. Temperature and rainfall capture environmental influences on virus viability, human behavior (indoor vs. outdoor activities), and respiratory tract susceptibility [51, 52]. Previous studies have shown that lower temperatures and higher humidity can enhance coronavirus survival on surfaces and in aerosols, potentially increasing transmission risk [52]. Our model's ability to incorporate these meteorological patterns enables weather‐adjusted forecasts, consistent with findings from Şahin [51], who demonstrated significant correlations between weather parameters and COVID‐19 spread.

Vaccination rate directly reduces susceptible population size and disease severity, fundamentally altering epidemic trajectories. The inclusion of this variable allows the model to capture the transition from pre‐vaccine to post‐vaccine epidemic dynamics, a critical feature for 2021 Malaysian data spanning the National COVID‐19 Immunization Program rollout. Tartof et al. [50] demonstrated that vaccine effectiveness varies over time, making vaccination rate a dynamic predictor that our LSTM component can model effectively.

Population density serves as a proxy for contact rates and transmission opportunities. Higher‐density areas typically experience faster disease spread, particularly relevant for Malaysia's diverse geography ranging from dense urban centers (Kuala Lumpur, Penang) to rural regions. Rader et al. [53] showed that crowding patterns significantly influence epidemic shapes, validating the inclusion of density as an exogenous predictor.

The dual integration of these variables at both the ARIMAX and LSTM stages enables comprehensive modeling consistent with hybrid statistical‐machine learning frameworks [54]. ARIMAX captures direct, linear relationships (e.g., vaccination rate linearly reducing cases), while LSTM models complex, nonlinear interactions (e.g., threshold effects where vaccination only reduces transmission substantially after reaching critical coverage) [46]. This architecture effectively addresses the multifaceted nature of infectious disease dynamics influenced by behavioral, environmental, and policy factor.

The proposed model successfully combines the complementary strengths of statistical and deep learning methods while mitigating their individual weaknesses [54]. The ARIMA model Captures stable linear trends, handles stationarity through differencing, provides interpretable coefficients for external variables, and requires relatively little training data. Our ARIMA (2,2,4) specification achieved adequate fit (AIC = 3455.298) with no residual autocorrelation (Ljung‐Box *Q* = 0.03, *p* = 0.86) [55], confirming proper model identification. The LSTM models complex nonlinear relationships and captured long‐term temporal dependencies through memory cells [56]. The LSTM's ability to process sequential information makes it ideal for capturing epidemic waves and transition phases. Our architecture with 64 units and 20% dropout balances representational capacity against overfitting risk.

In the hybrid architecture, decomposing the forecasting problem into linear and nonlinear components, the hybrid architecture allocates each model to its area of strength. ARIMAX handles predictable, trend‐based components while LSTM focuses on residual patterns containing complex dynamics [57]. This residual‐based approach is particularly effective: rather than forcing LSTM to learn everything from scratch, it focuses on correcting ARIMAX errors, which reduces learning burden, accelerates convergence, and improves generalization to unseen data explaining why the proposed model‐maintained accuracy during the critical September 2021 evaluation period. This approach aligns with ensemble learning principles where diverse models complement each other's weaknesses [58, 59].

Although this study focuses on Malaysia, the hybrid ARIMAX‐LSTM framework is structurally generalizable to other settings and diseases, as it separates linear epidemiological trends from nonlinear dynamics in a way that is not country‐specific. It can be applied elsewhere given comparable incidence data and relevant external drivers. However, recalibration with local data is necessary, as performance may vary due to differences in surveillance systems, interventions, and population behavior. The strong results observed in Malaysia nonetheless suggest that hybrid statistical–deep learning approaches represent a broadly applicable strategy for epidemic forecasting.

Regarding the validation strategy, this study relies on a single chronological train‐test split, which preserves temporal ordering and avoids data leakage—a standard and appropriate approach for time‐series epidemic forecasting. While this design is well‐suited to the sequential nature of the data, it does not fully capture model stability across varying epidemic phases. Future work should consider rolling‐window or walk‐forward cross‐validation schemes to more rigorously assess generalizability across different time periods and epidemic waves, further strengthening confidence in the model's robustness.

## 6 Limitations

While the findings are promising, several limitations must be acknowledged. First, the model was trained on Malaysian data from January–August 2021, limiting its generalizability to other regions and more recent viral phases such as Omicron. Second, incorporating additional exogenous variables such as testing rates, healthcare capacity, and socioeconomic indicators could further improve predictive accuracy. Third, the framework produces only deterministic point forecasts without uncertainty quantification, and future work should extend it to probabilistic approaches such as Bayesian LSTM or quantile regression to generate calibrated prediction intervals. Fourth, the model is currently limited to one‐step‐ahead predictions, and extensions to multi‐step forecasting horizons of 7, 14, or 30 days represent an important future direction. Finally, the interpretability of the LSTM component remains constrained by its black‐box nature, and the integration of attention mechanisms or explainable AI techniques is recommended to enhance transparency and user trust. Although Diebold–Mariano statistical tests provide empirical and statistical evidence of forecast reliability, the proposed deterministic hybrid framework does not explicitly generate probabilistic prediction intervals. Future work could extend the model to probabilistic forecasting using bootstrap residual simulation or Bayesian deep‐learning approaches.

A notable methodological limitation of this study is the reliance on a single chronological train–validation–test split over a 258‐day observation period. While this design preserves temporal integrity and prevents data leakage, it does not permit assessment of model robustness across multiple forecast origins. Future studies with longer time series spanning multiple epidemic waves, or multi‐country datasets, should employ rolling‐origin or walk‐forward validation to more comprehensively characterize the generalizability of hybrid ARIMAX‐LSTM forecasting frameworks.

## 7 Conclusions

This study developed and evaluated a hybrid ARIMA‐LSTM forecasting framework incorporating exogenous variables for COVID‐19 case prediction in Malaysia. By integrating four national‐level covariates‐daily average temperature, rainfall, vaccination rate, and population density at both the linear (ARIMAX) and nonlinear (LSTM residual) stages, the proposed dual‐stage exogenous integration strategy achieved substantial improvements overall baseline models on the test dataset (August 11–September 18, 2021), attaining *R* ^2^ = 0.7883, MAPE = 6.61%, RMSE = 948.62, and MAE = 769.49.

These results demonstrate that incorporating domain‐relevant exogenous variables within a hybrid framework yields meaningful gains in predictive accuracy compared to standalone ARIMA (*R* ^2^ = 0.046), standalone LSTM (*R* ^2^ = 0.22), and a standard hybrid ARIMA‐LSTM without exogenous inputs (*R* ^2^ = 0.0483). Notably, the standard hybrid model without exogenous variables provided only marginal improvement over standalone ARIMA, confirming that domain‐informed design rather than model complexity alone is the primary driver of forecasting performance. The proposed model also demonstrated practical efficiency, requiring 4–5 min of training and approximately 500 ms of inference time on consumer‐grade hardware.

It must be acknowledged, however, that these findings are based on a single national‐level dataset spanning one epidemic wave in Malaysia (January–September 2021). Accordingly, conclusions regarding generalizability to other countries, epidemic phases, or demographic contexts should be drawn cautiously. The model's performance under different viral variants, healthcare systems, or data availability conditions remains to be established. Similarly, while the results suggest potential utility for public health forecasting, direct policy application would require further validation across broader contexts before operational deployment.

The key contributions of this study are: (1) demonstrating the added predictive value of dual‐stage exogenous integration within a hybrid ARIMA‐LSTM architecture; (2) providing a reproducible, computationally efficient framework for national‐level epidemic forecasting; and (3) establishing a baseline for future hybrid forecasting research in the Malaysian and Southeast Asian context.

Future work should address the current limitations by expanding validation to sub‐national levels and across multiple epidemic waves, incorporating explicit policy intervention covariates such as Movement Control Order phases, integrating mobility data as temporal variables, developing multi‐step ahead forecasting capabilities, and enhancing model interpretability through explainable AI techniques. Exploring uncertainty quantification methods compatible with the hybrid architecture such as Monte Carlo dropout or ensemble‐based approaches would also strengthen the reliability of forecast outputs for public health decision‐making.

## Author Contributions

A.M. and M.A.A.N. contributed to the conceptualization, design of the study, data collection and formal analysis. K.I.M., F.M.H., and Z.M.Y.B. contributed to the interpretation of the study, and critical revision of the manuscript. All authors approved the submission of the final version of the manuscript.

## Disclosure

All authors have read and approved the final version of the manuscript. The corresponding authors, Al Mahmud and Mohamad Arif Awang Nawi, had full access to all the data in the study and take responsibility for the integrity of the data and the accuracy of the data analysis.

## Ethics Statement

The authors have nothing to report.

## Consent

The authors have nothing to report.

## Conflicts of Interest

The authors declare no conflicts of interest.

## Transparency Statement

Al Mahmud and Mohamad Arif Awang Nawi (corresponding authors) affirm that this manuscript is an honest, accurate, and transparent account of the study being reported; that no important aspects of the study have been omitted; and that any discrepancies from the study as planned (and, if relevant, registered) have been explained.

## Supporting information

Supporting File 1

## Data Availability

The data that support the findings of this study are available from the corresponding authors upon reasonable request.
