# Supplementary material for: Enhancing COVID‐19 Forecasting Accuracy in Malaysia Using a Hybrid ARIMA‐LSTM Model With Exogenous Variables: A Time‐Series Predictive Study
Source: Health Sci Rep. 2026 Jun 22;9(6):e72684. doi: 10.1002/hsr2.72684 (PMC13284900; doi:10.1002/hsr2.72684)
Supplement: Supplementary file 1 — Supporting File 1 [file HSR2-9-e72684-s001.docx]

**Supplementary Table 1: Model Diagnostics of ARIMA (6, 1, 6) Parameter.**

| Model Diagnostics of ARIMA (6, 1, 6) | |
| --- | --- |
| **Statistic** | **Value** |
| AIC | 3455.298 |
| BIC | 3499.297 |
| HQIC | 3473.07 |
| Log Likelihood | −1714.649 |
| Ljung-Box (lag 1, Q) | 0.03 (p = 0.86) |
| Jarque-Bera Test (JB) | 33.49 (p < 0.001) |
| Heteroskedasticity (H) | 2.42 (p < 0.001) |
| Skewness | 0.3 |
| Kurtosis | 4.82 |

**Supplementary Table 2: Model Estimation Results of ARIMA (6, 1, 6) Parameter.**

| **ARIMA (6, 1, 6) Model Estimation Results** | | | | | |
| --- | --- | --- | --- | --- | --- |
| **Parameter** | **Coefficient** | **Std. Error** | **z-value** | **P -value** | **95% CI** |
| AR (1) | 1.1433 | 0.144 | 7.924 | P = < 0.001 | [0.861, 1.426] |
| AR (2) | −0.0756 | 0.152 | −0.498 | P = 0.61 | [−0.373, 0.222] |
| AR (3) | −0.4536 | 0.132 | −3.433 | P= 0.001 | [−0.713, −0.195] |
| AR (4) | −0.4442 | 0.156 | −2.852 | P= 0.004 | [−0.749, −0.139] |
| AR (5) | 1.1851 | 0.139 | 8.533 | P = < 0.001 | [0.913, 1.457] |
| AR (6) | −0.4456 | 0.143 | −3.121 | P = 0.002 | [−0.725, −0.166] |
| MA (1) | −1.3783 | 0.144 | −9.585 | P= < 0.001 | [−1.660, −1.096] |
| MA (2) | 0.2831 | 0.192 | 1.475 | P = 0.14 | [−0.093, 0.659] |
| MA (3) | 0.4428 | 0.108 | 4.091 | P= < 0.001 | [0.231, 0.655] |
| MA (4) | 0.4349 | 0.207 | 2.105 | P = 0.035 | [0.030, 0.840] |
| MA (5) | −1.4080 | 0.194 | −7.248 | P= < 0.001 | [−1.789, −1.027] |
| MA (6) | 0.7877 | 0.117 | 6.722 | P= < 0.001 | [0.558, 1.017] |

**Supplementary Table 3: Residual Diagnostic Test of ARIMA (6, 1, 6) Model.**

| Residual Diagnostic Tests for ARIMA (6, 1, 6) Model | | | |
| --- | --- | --- | --- |
| **Test** | **Value** | **P -value** | **Interpretation** |
| Ljung–Box Q (lag 1) | 0.03 | P = 0.86 | No autocorrelation in residuals (good) |
| Jarque–Bera (JB) | 33.49 | P < 0.001 | Residuals not normally distributed |
| Heteroskedasticity (H test) | 2.42 | P < 0.001 | Residuals show non-constant variance |
| Skewness | 0.3 | – | Slight right skew |
| Kurtosis | 4.82 | – | Heavy-tailed residuals |

**Supplementary Table 4: LSTM Model parameter**

| **Parameter** | **Value** |
| --- | --- |
| LSTM Units | 200 |
| Batch Units | 200 |
| Epochs | 16 |
| Verbose | 1 |
| Optimization Approach | Adam |

**Supplementary Table 5: ARIMA (6, 1, 6) Model Performance**

| ARIMA Model | |
| --- | --- |
| **Metric** | **Value** |
| Mean Squared Error (MSE) | 3,451,941.18 |
| Root Mean Squared Error (RMSE) | 1,857.94 |
| Mean Absolute Error (MAE) | 1,537.18 |
| Mean Absolute Percentage Error (MAPE) | 7.55% |
| R-squared (R²) | 0.046 |

**Supplementary Table 6: LSTM Model Performance**

| LSTM Model | |
| --- | --- |
| **Metric** | **Value** |
| Mean Squared Error (MSE) | 3,738,848.24 |
| Root Mean Squared Error (RMSE) | 1,933.60 |
| Mean Absolute Error (MAE) | 1,713.36 |
| Mean Absolute Percentage Error (MAPE) | 9.42% |
| R-squared (R²) | 0.220 |

**Supplementary Table 7: Hybrid ARIMA-LSTM Model Performance**

| **Hybrid ARIMA-LSTM** |  |
| --- | --- |
| **Metric** | **Value** |
| Mean Squared Error (MSE) | 3,443,556.74 |
| Root Mean Squared Error (RMSE) | 1,855.68 |
| Mean Absolute Error (MAE) | 1,535.44 |
| Mean Absolute Percentage Error (MAPE) | 7.55% |
| R-squared (R²) | 0.0483 |
